# Appropriate Parenting Style to Improve Offspring's Creativity Differs Depending on the Offspring's Empathizing–Systemizing Cognitive Style: A Voxel-Based Morphometry Study

**DOI:** 10.1155/bn/8156740

**Published:** 2025-04-21

**Authors:** Radiztia Ekayantri Zulkifli, Yasuko Tatewaki, Hikaru Takeuchi, Diptarama Hendrian, Ryuta Kawashima, Yasuyuki Taki

**Affiliations:** ^1^Department of Aging Research and Geriatric Medicine, Institute of Development, Aging and Cancer, Tohoku University, Sendai, Japan; ^2^Division of Developmental Cognitive Neuroscience, Institute of Development, Aging and Cancer, Tohoku University, Sendai, Japan; ^3^Graduate School of Information Sciences, Tohoku University, Sendai, Japan; ^4^M&D Data Science Center, Institute of Science Tokyo, Tokyo, Japan; ^5^Smart-Aging Research Center, Tohoku University, Sendai, Japan

**Keywords:** adolescence, brain development, creativity, empathizing–systemizing cognitive style, offspring, parenting style, supramarginal gyrus

## Abstract

Previous research by Mehrinejad et al. found that parenting style affects offspring's brain development which later determines their creativity. They show that there is a significant positive relationship between authoritative parenting style and creativity. Meanwhile, neuroimaging studies by Takeuchi et al. have explained that offspring's creativity also differs depending on their empathizing–systemizing cognitive style. They show that both EQ and SQ were significantly and positively correlated with creativity. Combining the previous studies, we hypothesize that there exists an effect of the positive interaction between parenting style and the offspring's E-S cognitive style on the offspring's brain development and creativity. Whereas previous research on the offspring's creativity has focused on one dimension only, in the current study, for the first time, we investigated the effect of the interaction between parenting style and the offspring's E-S cognitive style on the offspring's brain development and creativity. We used voxel-based morphometry and questionnaires to investigate the gray matter correlates of the interaction between parenting style and the offspring's E-S cognitive style. With 675 healthy participants (average: 20 years old), using cross-sectional whole-brain multiple regression, we found significant interactive effects between parenting style and offspring's E-S cognitive style on regional gray matter volume (rGMV) in the right supramarginal gyrus (R-SMG). We also found that the rGMV in the R-SMG was significantly and positively correlated with the offspring's originality/fluency scores, a dimension of creativity. Our findings suggest that offsprings who are raised with the appropriate parenting style according to their E-S cognitive style have a larger rGMV in the R-SMG. These offsprings exhibit a higher level of creativity, especially originality in divergent thinking, the ability to generate an idea that is different from that of others. In the future, we hope this research can provide critical information for parents regarding the parenting style that suits their offspring's E-S cognitive style to improve offspring's creativity and quality of life.

## 1. Introduction

Creativity is the ability to generate novel and valuable ideas, solutions, or products that are relevant within a certain social or cultural context [1, 2]. Divergent thinking (DT) is the ability to generate various ideas by exploring multiple possible solutions or perspectives on open-ended problem [3]. Previous research by Guilford [4] revealed that DT is the core of creativity. DT tests have become the most popular psychometric assessment tool for evaluating creativity [5] because of their high test reliability and validity [6].

Mehrinejad et al. [7] found that parenting style (child-rearing) can nurture offspring's creativity. The emotional bonding, which is formed by the quality of parenting style, shows a significant impact on the offspring's learning and development process [8].

On the other hand, a neuroimaging study from Takeuchi et al. [9] explained that an individual's creativity differs depending on their empathizing–systemizing (E-S) cognitive style. They show that the score of both empathizing quotient (EQ) and systemizing quotient (SQ) is significantly and positively correlated with creativity.

These studies led to a question: To improve offspring's brain development and creativity, do parents need to adapt their parenting style to their offspring's E-S cognitive style? Our goal is to investigate whether there is any interaction between parenting style and offspring's E-S cognitive style on the offspring's brain development and their creativity. If an interaction exists, we can determine what kind of parenting style which is most appropriate for each offspring's E-S cognitive style. We hypothesize that there exists an effect of the positive interaction between parenting style and the offspring's E-S cognitive style on the offspring's brain development and creativity. In the future, we hope this research can provide critical information for parents regarding the parenting style that suits their offspring's E-S cognitive style to improve offspring's creativity and quality of life.

Firstly, to evaluate offspring's brain development, we employed voxel-based morphometry (VBM) [10] rather than region of interest (ROI) approach. ROI approach is limited to selected regions [11]. On the contrast, VBM is a fully automated technique for examining the entire brain [12]. VBM can capture regional differences in gray or white matter that are not observable using the ROI method [13]. Moreover, the small number of participants was also a problem in previous Japanese studies (*N* = 50) [14]. We recruited 675 participants to meet the requirements for statistical adequacy to conduct this research.

Secondly, we used DT tests to evaluate offspring's creativity. DT tests consist of four subscales: fluency, flexibility, originality, and elaboration. Fluency is the ability to respond to a question with a relevant answer and to produce several alternative answers. Flexibility is the ability to produce responses from a broad perspective. Originality is the ability to produce ideas that differ from those of others. Elaboration is the ability to produce detailed ideas [15]. However, separate interpretation of DT subscale scores is challenging [16] because of strong correlations among subscale scores when scoring with traditional methods [17]. We used originality/fluency score in the present study, in accordance with previous studies by Eisenman [18] and Takeuchi et al. [19].

Thirdly, we used the Parental Bonding Instrument (PBI) to quantitatively assess parenting style. The PBI is a self-reported questionnaire to describe separately memoirs of the fathers' and mothers' parenting style from an offspring's perspective in the first 16 years of their life [20]. The reliability and validity of the PBI have been confirmed and used in clinical research [21]. The PBI has two subscales: “care (CA)” refers to father/mother's love, acceptance, and sensitiveness to offspring's emotional and developmental needs; “overprotection (OP)” refers to father/mother's control over the offspring's behavior and decisions. Because the authors focused on assessing parenting style in one family, in this study, we added the father's CA score and the mother's CA score as the parent's CA score, as well as the father's OP score and the mother's OP score as the parent's OP score. Internal consistency values demonstrated the reliability of this combined score (*α* = 0.92 and 0.87, respectively). We used normalized *Z* scores of parent's CA denoted as *Z*_*CA*_ and normalized *Z* scores of parent's OP denoted as *Z*_*OP*_. Moreover, we used the difference between the *Z*_*CA*_ and *Z*_*OP*_ denoted as *D*_*CA*−*OP*_, to evaluate the balance between CA and OP of the parents.

Finally, offspring's E-S cognitive style is evaluated by the EQ and SQ questionnaires [22]. The EQ measures the ability to feel and understand another person's emotions appropriately, and the SQ measures the ability to analyze and construct systems. E-S theory categorizes people into five categories: Extreme Type E (EQ > > SQ), Type E (EQ > SQ), Type B (EQ = SQ), Type S (SQ > EQ), and Extreme Type S (SQ > > EQ). Furthermore, Wakabayashi [23] found no developmental changes in E-S cognitive styles. A person's E-S cognitive style is determined prenatally rather than postnatally, therefore does not show any significant change from childhood until adulthood.

## 2. Materials and Methods

### 2.1. Participants

The participants were healthy Japanese young adults. Data from 685 participants were used in this study as part of an ongoing project aimed at investigating associations among brain imaging, cognitive function, aging, genetics, and daily habits [24]. Furthermore, 10 participants were excluded because they were identified as outliers using maximum *Z* approach [25, 26]. Finally, 675 participants (377 males and 298 females; mean age = 20.86 ± 1.59) were included in the present study.

Participants had no history of neurological or psychiatric disorders, and their right-handedness was evaluated using the Edinburgh Handedness Inventory [27].

### 2.2. Ethical Considerations

This study was approved by the institutional review board of Tohoku University and conducted in accordance with the Declaration of Helsinki (1991). Written informed consent was obtained from all participants.

### 2.3. Psychological Measures

#### 2.3.1. PBI

We administered the Japanese version of PBI, a self-reported questionnaire for assessing parenting style. Participants were instructed to answer each question with recollections of their parents' parenting style in their first 16 years of life. The PBI has two subscales: CA and OP. Twelve questions were about “CA” (e.g., “was affectionate to me” and “seemed emotionally cold to me” [reversed]). Thirteen questions were about “OP” (e.g., “trying to control everything I did” or “let me decide things for myself” [reversed]).

The scales consist of self-descriptive statements scored on a 4-point scale (0–3), ranging from *strongly disagree* to *strongly agree*. In this study, maternal and paternal composites showed high internal consistency for CA (*α* = 0.92 and 0.88, respectively) and OP (*α* = 0.82 and 0.87, respectively). In accordance with a previous study [28], we used total CA and OP scores for parenting style to investigate the interactive effect by adding maternal and paternal scores (*α* = 0.92 and 0.87, respectively). Internal consistency values demonstrated the reliability of this questionnaire.

To assess parenting style, we used normalized *Z* scores of CA denoted as *Z*_*CA*_, normalized *Z* scores of OP denoted as *Z*_*OP*_, and the difference between *Z*_*CA*_ and *Z*_*OP*_ denoted as *D*_*CA*−*OP*_. The *Z* score of CA can be obtained by subtracting the sample mean from the score and then dividing the result by the sample standard deviation: *Z*_*CA*_ = (*CA* − *μ*_*CA*_)/*σ*_*CA*_, where *CA* is the raw CA scores, *μ*_*CA*_ is the sample mean of the raw CA score, and *σ*_*CA*_ is the sample standard deviation of the raw CA score. Similarly, the *Z* score for OP can be calculated by *Z*_*OP*_ = (*OP* − *μ*_*OP*_)/*σ*_*OP*_. The discrepancy between CA and OP was then quantified as *D*_*CA*−*OP*_ = (*Z*_*CA*_ − *Z*_*OP*_)/2. The greater the *D*_*CA*−*OP*_ score in a positive direction, the stronger parent's care is relative to the parent's overprotection. *D*_*CA*−*OP*_ score close to zero represents an equal drive to care for and overprotect the offspring. The greater the *D*_*CA*−*OP*_ score in a negative direction, the stronger parent's overprotection is relative to the parent's care.

#### 2.3.2. SQ and EQ Questionnaires

We use the same methods from previous study [9] for assessing E-S cognitive style. We administered the Japanese version [29] of the SQ/EQ questionnaire [30, 31] for assessing each offspring's E-S cognitive style. The SQ score was used as an index of systemizing and the EQ score was used as an index of empathizing. Each questionnaire consists of 60 items, that is, 40 items assessing SQ (respectively, EQ) and 20 unscored filler items. The items consist of self-descriptive statements that are scored on a 4-point scale: “*strongly disagree*,” “*slightly agree*,” “*slightly disagree*,” and “*strongly disagree*.” Half of the nonfiller items are worded to produce an “*agree*” response, which “*strongly agree*” responses scored 2 points, “*slightly agree*” responses scored 1 point, and other responses scored 0 point. The other half of the nonfiller items are worded to produce a “*disagree*” response which “*strongly disagree*” responses scored 2 points, “*slightly disagree*” responses scored 1 point, and other responses scored 0 point, thus yielding a range of total scores between 0 and 80 for each quotient. Items are randomized to avoid response bias.

The following are examples of items included in the SQ–EQ questionnaire:

“I am fascinated by how machines work.” (SQ)

“If I were buying a stereo, I would want to know about its precise technical features.” (SQ)

“I can tune into how someone else feels rapidly and intuitively.” (EQ)

“I am good at predicting how someone will feel.” (EQ)

The internal consistencies of EQ and SQ, calculated in a previous study [29] that included a large sample, were 0.86 and 0.88, respectively, demonstrating the reliability of this questionnaire.

The *D*_*SQ*−*EQ*_ score was calculated in a similar way as *D*_*CA*−*OP*_. Raw SQ and EQ scores were standardized by using *Z* scores which can be obtained by subtracting the sample mean from the score and then dividing the result by the sample standard deviation score: *Z*_*SQ*_ = (*SQ* − *μ*_*SQ*_)/*σ*_*SQ*_ and *Z*_*EQ*_ = (*EQ* − *μ*_*EQ*_)/*σ*_*EQ*_. The discrepancy between systemizing and empathizing was then quantified as *D*_*SQ*−*EQ*_ = (*Z*_*SQ*_ − *Z*_*EQ*_)/2. The greater the *D*_*SQ*−*EQ*_ score in a positive direction, the stronger an individual's systemizing is relative to their empathizing. The *D*_*SQ*−*EQ*_ score close to zero represents an equal drive to systemize and empathize. The greater the *D*_*SQ*−*EQ*_ score in a negative direction, the stronger the individual's empathizing is relative to their systemizing.

### 2.4. Behavioral Data

#### 2.4.1. Assessment of Offspring's Originality in Creativity Measured by Divergent Thinking (CMDT)

We use the same methods from previous study [9] for assessing creativity. The S-A creativity test [15] was used for assessing CMDT. Guilford [4] generated the draft plan of this test and supervised the development of the test [15]. The test was standardized for Japanese speakers [15].

The test is used for evaluating verbal CMDT [15], and it involves three types of tasks. This test was administered in a group setting. Each task consists of 2 min of practice and 5 min of task with two questions. Practice and tasks were administered in the following order: (1) practice of the first task (2 min), (2) the first task (5 min), (3) practice of the second task (2 min), (4) the second task (5 min), (5) practice of the third task (2 min), and (6) the third task (5 min). In total, the test takes 30 min. How subjects divided their time (5 min in total) for the two questions was not determined.

The first task requires subjects to generate unique ways of using typical objects (e.g., “Other than for drinking milk, how can we use milk bottles?” Example answer: “We can use them as containers.”). The second task requires subjects to imagine desirable functions of ordinary objects (e.g., “What are the characteristics of a good TV? Write down as many characteristics as possible.” Example answer: “A TV can receive broadcasts from all over the world.”). The third task requires subjects to imagine the consequences of “unimaginable things” happening (e.g., “What would happen if all the mice in the world disappeared?” Example answer: “The world would become more hygienic.”). For each task, subjects are required to generate as many answers as possible. Note that these tasks correspond to the three tasks (unusual use, product improvement, just suppose) of the Torrance test of creative thinking [17], which is used in other countries.

Scoring was performed by the Tokyo Shinri Corporation. In addition to a total score, the S-A creativity test provides subscores for the following dimensions of creativity: (a) Fluency is the ability to produce and consider several alternatives. Fluency scores are determined by the total number of questions answered after excluding inappropriate responses or responses that are difficult to understand. (b) Flexibility is the ability to produce responses from a broad perspective. Flexibility scores are determined by the sum of the (total) number of category types to which the responses were assigned based on a criteria table or similar judgment. (c) Originality is the ability to produce ideas that differ from those of others. For originality scoring, each answer was assigned to an idea category from a criteria table or similar judgment. Each category received different originality points on the basis of appearance frequency, and the originality scores were calculated as the sum of all of these points. In the case of the first task, answers were categorized into “containers” had high appearance frequencies (> 5%) and were thus awarded 0 points. Alternatively, the answers categorized as “alternatives for musical instruments” had lower appearance frequencies (1%–5%) and were thus awarded 1 point, whereas rarer answer categories or answers that could not be categorized were awarded 2 points. (d) Elaboration is the ability to produce detailed ideas. Elaboration scores are determined by the sum of responses weighted based on a criteria table or similar judgment. In the case of the first task, answers that were classified as the lowest level of elaborateness, “unclear answers” such as “musical instruments” (within the “alternatives for musical instruments” category), were awarded 0 points, while answers classified to the middle level of elaborateness which have typically only means or purposes such as “beat and make sounds” were awarded 1 point, and answers classified as the highest level of elaborateness which have typically both means and purposes and/or more details such as “arrange milk bottles in a row and put different amounts of water in each bottle and beat to use as instruments” were awarded 2 points. Again, these four dimensions correspond to those of a previous study [17].

In the present study, originality/fluency scores were examined. Strong correlations were noted among fluency, elaboration, and flexibility (*r* > 0.78 in this study), while originality scores exhibited a distinctive pattern, with simple correlation coefficients of 0.54–0.70, consistent with the distinctive psychological characteristic of the originality/fluency score. Originality/fluency score has been used by other researchers [18] and represents the originality of answers after adjusting for the number of responses (infrequency of each generated idea). Because the originality score itself is the sum of the originality score of each generated idea, it is directly affected by fluency. Therefore, an originality/fluency score was used in this study, consistent with previous research [32]. This score shows a substantial correlation with originality but little correlation with fluency (meaning this score reflects components specific to originality and does not reflect components specific to fluency), thereby simplifying interpretation.

#### 2.4.2. Assessment of Psychometric Measures of General Intelligence

We use the same methods from previous study [9] for assessing psychometric measures of general intelligence. Raven's Advanced Progressive Matrix (RAPM) [33] is one of the most specific psychometric measures of general intelligence [33]. Because the RAPM has been reported to be the most strongly correlated with general intelligence and is the most widely used measure of general intelligence [33], we used it to assess intelligence in the current study. This test was used in the current study to adjust for the effects of individual psychometric measures of intelligence on brain structure. This adjustment was performed because creativity is known to be associated with psychometric measures of intelligence among subjects of low to average intelligence [34]; thus, we did not necessarily expect to find a significant correlation in our sample of highly educated subjects. This adjustment was also performed to exclude the possibility that any significant correlation between regional gray matter volume (rGMV) and creativity was caused by the indirect association between rGMV and general intelligence, which is a higher-level cognitive function than creativity. The RAPM [33] contains 36 nonverbal items requiring fluid reasoning ability. Each item consists of a 3 × 3 matrix with a missing piece to be completed by selecting the best of eight alternatives. The score of this test (the number of correct answers in 30 min) was used as an index of individual psychometric measures of intelligence.

### 2.5. Image Acquisition

We use the same methods from previous study [35] for the image acquisition. All brain magnetic resonance images (MRIs) were collected using a 3-T Intera Achieva scanner. Three-dimensional high-resolution T1-weighted structural images were collected by using a magnetization-prepared rapid gradient echo sequence. The imaging parameters were as follows: matrix, 240 × 240; repetition time, 6.5 ms; echo time, 3 ms; field of view, 24 cm; slices, 162; and slice thickness, 1.0 mm.

### 2.6. Image Preprocessing

Structural MRI data were analyzed using Statistical Parametric Mapping software (SPM12; Welcome Department of Cognitive Neurology, London, United Kingdom) implemented in MATLAB (MathWorks, Inc., Natick, Massachusetts). Preprocessing entailed the following four main steps. First, T1-weighted structural images were reoriented using an automated reorienting script (https://www.nemotos.net/?p=1892) in MATLAB. Second, the reoriented T1-weighted structural images from each participant were segmented into six tissues, including gray matter, white matter, cerebrospinal fluid, soft tissue, skull, and nonbrain regions using the new segmentation algorithm implemented in SPM12. The default parameters were used, except for affine regularization, which was performed using the International Consortium for Brain Mapping (ICBM) template for East Asian brains. After segmentation, we conducted the Diffeomorphic Anatomical Registration Through Exponentiated Lie Algebra (DARTEL) registration process to spatially normalize the tissue probability maps obtained by the abovementioned method on Montreal Neurological Institute (MNI) space. This yielded images with 1.5 × 1.5 × 1.5 mm^3^ voxels. Subsequently, all images were smoothed via convolution with an isotropic Gaussian kernel of 8-mm full width at half maximum. The total intracranial volume (TIV) was calculated by combining the total volume of the gray matter, white matter, and cerebrospinal fluid.

### 2.7. Statistical Analysis

#### 2.7.1. Interactive Effects of Parenting Style and Offspring's E-S Cognitive Style on rGMV

Before examining the interactive effects, we examined the independent effects of parenting style and offspring's E-S cognitive style on brain structure using the SPM12 software. We performed two whole-brain analyses to assess differences in rGMV. First, we conducted a whole-brain multiple regression analysis to examine the association between parenting style (*Z*_*CA*_, *Z*_*OP*_, *D*_*CA*−*OP*_) and rGMV. Second, we also ran a whole-brain multiple regression analysis to examine the association between an offspring's E-S cognitive style (*D*_*SQ*−*EQ*_) and rGMV. Participant's age, sex, TIV, and parent's annual income were added as covariates of no interest for both analyses. Statistical significance was considered achieved when the voxel fell below a peak level-corrected family wise error (*pFWE* *corr*.) value of 0.05.

Furthermore, to examine the interactive effects of parenting style and offspring's E-S cognitive style on rGMV, a whole-brain multiple regression analysis was conducted using SPM12 software. The parenting style, offspring's E-S cognitive style (mean-centered), and an interaction term comprising parenting style × offspring's E-S cognitive style were entered as predictor variables. Participants' age, sex, TIV, and parent's annual income were entered as covariates of no interest. Interaction terms between predictor variables and covariates of no interest (parenting style × age, parenting style × sex, parenting style × TIV, parenting style × parent's annual income, offspring's E-S cognitive style × age, offspring's E-S cognitive style × sex, offspring's E-S cognitive style × TIV, and offspring's E-S cognitive style × parent's annual income) were also entered as covariates of no interest to accurately control for confounders [36]. All continuous variables were mean centered before analysis. Voxels were considered significant when they fell below a peak level-corrected (*pFWE* *corr*.) value of 0.05.

For post hoc analysis, we extracted the mean voxel value of significant voxels from each participant using the eigenvariate option in the SPM12 software [37]. We performed post hoc analysis using the Johnson–Neyman method [38] to calculate the association between rGMV and parenting style × offspring's E-S cognitive style, and the values of offspring's E-S cognitive style in which rGMVs were significantly different among parenting style. The post hoc test was performed using a computational tool (http://www.quantpsy.org/interact/) developed by Preacher et al. [39], after the values necessary for analysis (regression coefficients and coefficients of variance of the intercept, parenting style, offspring's E-S cognitive style, and parenting style × offspring's E-S cognitive style, as well as coefficients of covariance between the intercept and parenting style and between offspring's E-S cognitive style and parenting style × offspring's E-S cognitive style) were calculated by multiple regression analysis using SPSS Statistics 24.0 (SPSS, Inc., Chicago, Illinois, United States).

#### 2.7.2. Correlation Between rGMV in the R-SMG (Resulting From the Interactive Effect Between Parenting Style and Offspring's E-S Cognitive Style) and Offspring's Originality of DT

Previous studies demonstrated neural evidence that the inferior parietal lobe (IPL), the subregions of which are the supramarginal gyrus (SMG) and the angular gyrus (AG), may contribute to the production of original ideas [40, 41]. On the basis of these previous studies, it is possible that rGMV in the R-SMG resulting from the interactive effect between *D*_*CA*−*OP*_ and *D*_*SQ*−*EQ*_ may correlate with the offspring's creativity, particularly in the dimension of originality.

For the correlation analysis, first we newly created the mask of R-SMG (Brodmann area 40) for ROI with WFU Pickatlas. WFU Pickatlas [42, 43] provides an atlas-based method for generating ROIs. Second, we calculate the rGMV in the R-SMG (resulting from the interactive effect between *D*_*CA*−*OP*_ and *D*_*SQ*−*EQ*_) using ROI analysis with the newly created mask and get_totals in Matlab. Third, we performed a partial correlational analysis with the index of creativity to investigate the relationships between the rGMV in the R-SMG (resulting from the interactive effect between *D*_*CA*−*OP*_ and *D*_*SQ*−*EQ*_) and the offspring's originality of DT (originality/fluency scores) using IBM SPSS Statistics 21 software. Participant's age, sex, parent's annual income, and RAPM general intelligence score were added as covariates. Pearson's correlation coefficient was used for the analysis, and *p* < 0.05 was used as the statistical significance threshold.

## 3. Results

### 3.1. Participants' Characteristics

**Figure 1:**
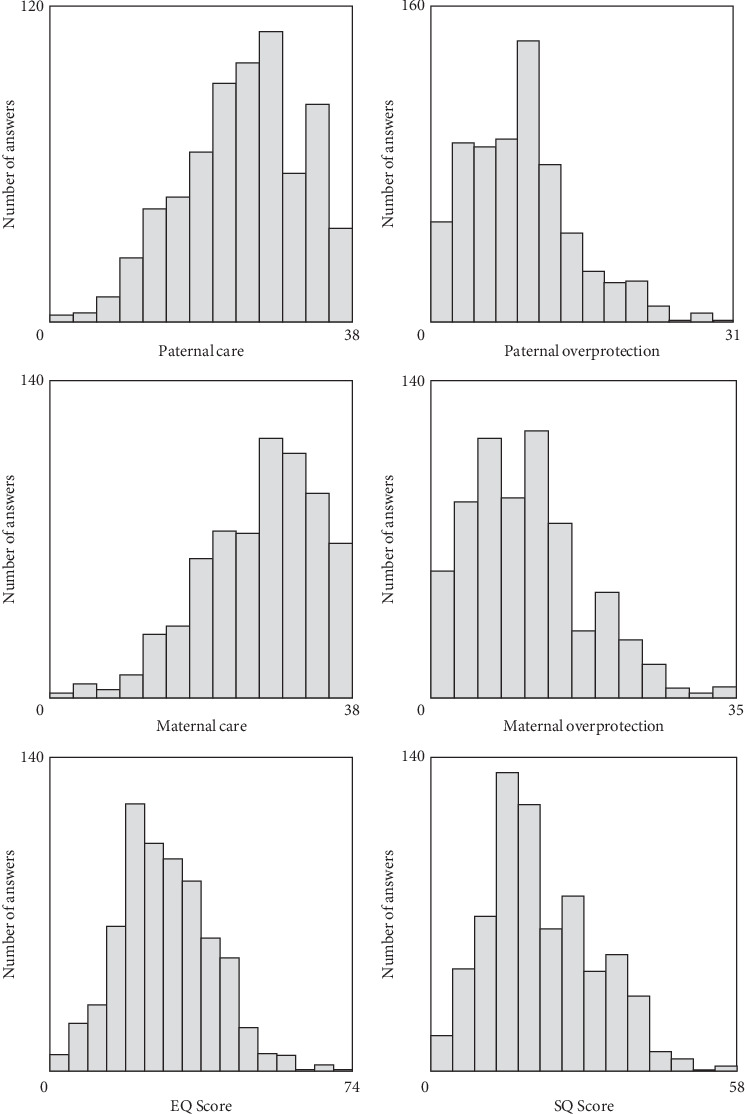
The distribution of paternal care, paternal overprotection, maternal care, and maternal overprotection based on published cut-off [20]. The kurtosis and skewness of all scores ranged from −1 to +1, which indicated the normality of data [28].

The distribution of paternal care, paternal overprotection, maternal care, maternal overprotection, offspring's EQ score, and offspring's SQ score is shown in Figure 1. The kurtosis and skewness of all scores ranged from −1 to +1, which indicated the normality of data [28].

### 3.2. Effects of Parenting Style (*Z*_*CA*_, *Z*_*OP*_, *D*_*CA*−*OP*_) on rGMV

Multiple regression analysis demonstrated no significant correlation between parenting style (*Z*_*CA*_, *Z*_*OP*_, *D*_*CA*−*OP*_) and rGMV in any region.

### 3.3. Effects of Offspring's E-S Cognitive Style (*D*_*SQ*−*EQ*_) on rGMV

**Figure 2:**
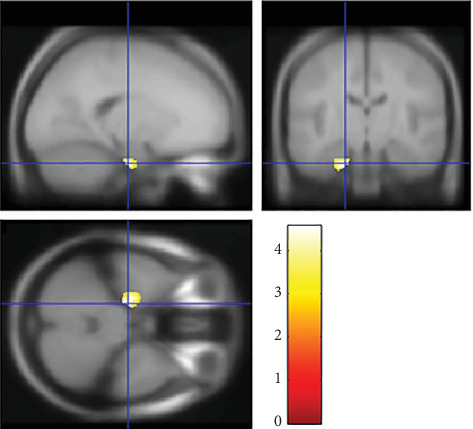
Brain areas in which the offspring's E-S cognitive style and rGMV in the left parahippocampal gyrus were significantly and positively correlated. MNI coordinates at peak voxel = [*x* = −18, *y* = −16, *z* = −32], *T* = 4.53, *p* = 0.039. Sex, age, parent's annual income, and offspring's total intracranial volume were entered as covariates of no interest. The left sides of the image represent the left side of the brain. The color bar indicates the *T* value. We adjusted the figure to account for the reduced statistical threshold to *p* < 0.001 uncorrected for visibility.

As shown in Figure 2, multiple regression analysis demonstrated a significant positive correlation between offspring's E-S cognitive style (*D*_*SQ*−*EQ*_) and rGMV in the left parahippocampal gyrus (MNI coordinates at peak voxel = (−18, −16, −32), *T* = 4.53, *p* = 0.039). However, there was no significant negative association between the offspring's E-S cognitive styles and rGMV in any region. Although there is a significant positive correlation between offspring's E-S cognitive style and rGMV in the left parahippocampal gyrus, since it is not related to the interaction between parenting style and offspring's E-S cognitive style, we do not discuss this result further.

### 3.4. Interactive Effects of the Parenting Style (*Z*_*CA*_, *Z*_*OP*_, *D*_*CA*−*OP*_) and Offspring's E-S Cognitive Style (*D*_*SQ*−*EQ*_) on rGMV

Multiple regression analysis using SPM12 demonstrated no significant interactive effect of the parenting style (*Z*_*CA*_, *Z*_*OP*_) and offspring's E-S cognitive style (*D*_*SQ*−*EQ*_) on rGMV in the any region.

**Figure 3:**
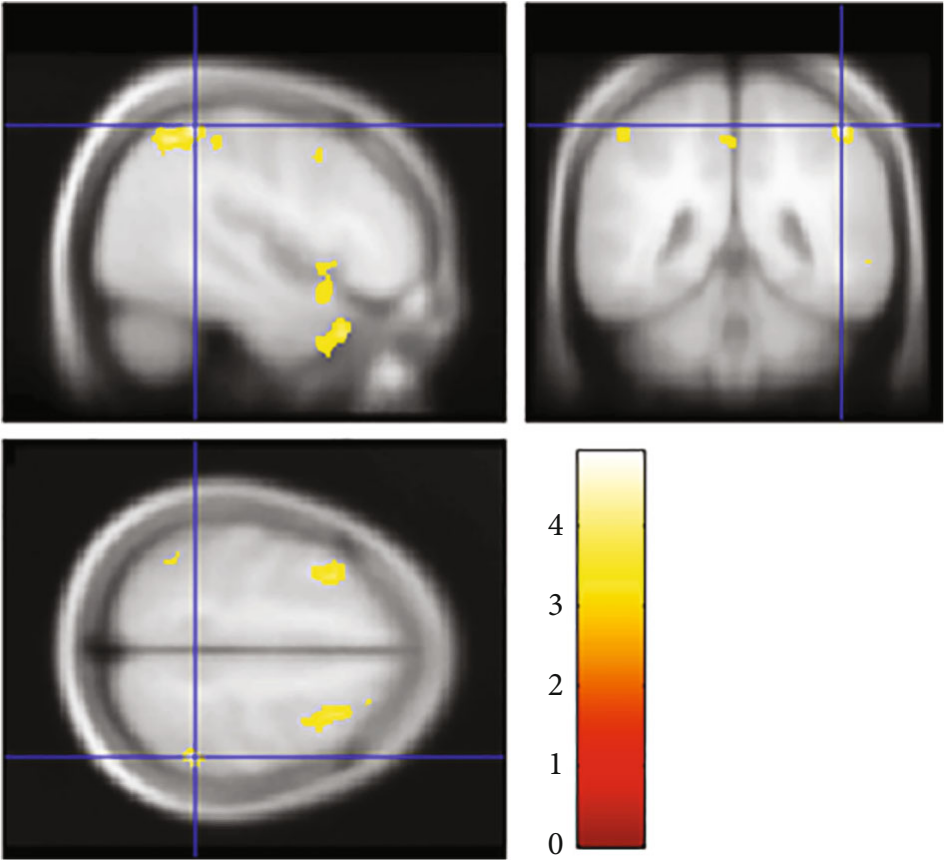
Interactive effect of parenting style and offspring's E-S cognitive style on rGMV in the R-SMG. Montreal Neurological Institute coordinates at peak voxel = [*x* = 46, *y* = −44, *z* = 56], *T* = 4.93, *p* = 0.009. Sex, age, parent's annual income, and offspring's total intracranial volume were entered as covariates of no interest. The left sides of the image represent the left side of the brain. The color bar indicates the *T* value. We adjusted the figure to account for the reduced statistical threshold to *p* < 0.001 uncorrected for visibility.

However, multiple regression analysis using SPM12 showed a significant interactive effect of parenting style (*D*_*CA*−*OP*_) and offspring's E-S cognitive style (*D*_*SQ*−*EQ*_) on rGMV in the R-SMG (Figure 3). Further analysis with Johnson–Neyman method shows that in range of *D*_*SQ*−*EQ*_ > 0.504, there was a significant positive correlation (*p* < 0.05) between *D*_*CA*−*OP*_ and rGMV in the R-SMG. In contrast, in range of *D*_*SQ*−*EQ*_ < −0.207, there was a significant negative correlation (*p* < 0.05) between *D*_*CA*−*OP*_ and rGMV in the R-SMG.

**Figure 4:**
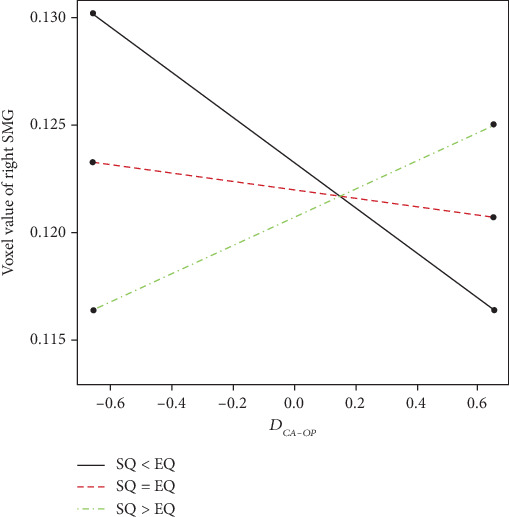
Interaction plot indicates that the association between parenting style and rGMV in the R-SMG was modulated by the offspring's E-S cognitive style. The black line indicates *D*_*SQ*−*EQ*_ < 0 or in other words when EQ score is higher than SQ score. The red line indicates *D*_*SQ*−*EQ*_ = 0 or in other words when EQ score is equal to SQ score. The green line indicates *D*_*SQ*−*EQ*_ > 0 or in other words when EQ score is lower than SQ score. Using Johnson–Neyman method, linear correlation of *D*_*CA*−*OP*_ and rGMV in the R-SMG, when *D*_*SQ*−*EQ*_ = 0.658 (+1*SD*), we have *rGMV* = 0.0647 + 0.0049∙*D*_*CA*−*OP*_ (*p* = 0.0154), and when *D*_*SQ*−*EQ*_ = −0.658 (−1*SD*), we have *rGMV* = 0.0673 − 0.0069∙*D*_*CA*−*OP*_ (*p* = 0.0004).

Figure 4 shows linear correlation of *D*_*CA*−*OP*_ and rGMV in the R-SMG. When *D*_*SQ*−*EQ*_ = 0.658 (+1*SD*), we have *rGMV* = 0.0647 + 0.0049∙*D*_*CA*−*OP*_ (*p* = 0.0154), and when *D*_*SQ*−*EQ*_ = −0.658 (−1*SD*), we have *rGMV* = 0.0673 − 0.0069∙*D*_*CA*−*OP*_ (*p* = 0.0004).

### 3.5. Correlation Between rGMV in the R-SMG (Resulting From the Interactive Effect Between Parenting Style and Offspring's E-S Cognitive Style) and the Offspring's Originality of DT

**Figure 5:**
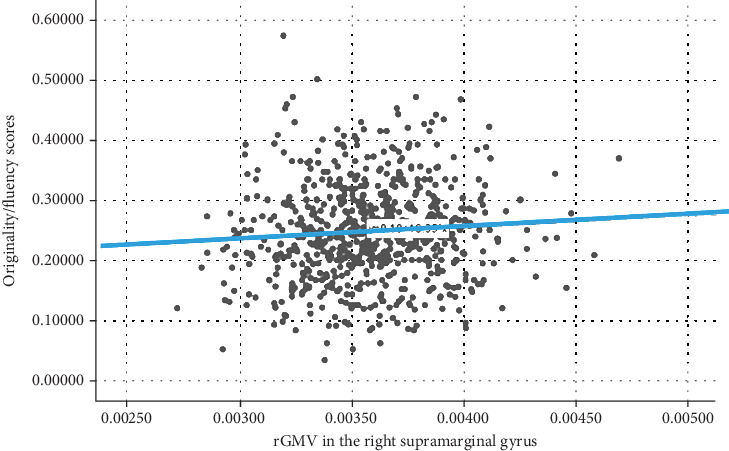
The scatter plot showed the association between rGMV in the R-SMG (resulting from the interactive effect between *D*_*CA*−*OP*_ and *D*_*SQ*−*EQ*_) and offspring's originality/fluency scores (*R* = 0.188; *p* = 0.008). Participant's sex, age, RAPM, and general intelligence score were added as covariates.

As shown in Figure 5, rGMV in the R-SMG (resulting from the interactive effect between *D*_*CA*−*OP*_ and *D*_*SQ*−*EQ*_) was significantly and positively correlated with the offspring's originality/fluency scores (*R* = 0.188; *p* = 0.008). Participant's age, sex, and RAPM general intelligence score were added as covariates.

## 4. Discussion

To the best of our knowledge, this is the first study to demonstrate an interactive effect between parenting style described as *D*_*CA*−*OP*_ and offspring's E-S cognitive style described as *D*_*SQ*−*EQ*_ on brain structure. The interaction between *D*_*CA*−*OP*_ and *D*_*SQ*−*EQ*_ significantly correlates with rGMV in the R-SMG. Further analysis with Johnson–Neyman method shows that in range of *D*_*SQ*−*EQ*_ > 0.504, there was a significant positive correlation (*p* < 0.05) between *D*_*CA*−*OP*_ and rGMV in the R-SMG. In contrast, in the range of *D*_*SQ*−*EQ*_ < −0.207, there was a significant negative correlation (*p* < 0.05) between *D*_*CA*−*OP*_ and rGMV in the R-SMG. We did not find a significant interaction between parenting style described as *Z*_*CA*_ or *Z*_*OP*_ and *D*_*SQ*−*EQ*_ on rGMV in any region. *D*_*CA*−*OP*_ represents the difference in parenting style of CA and OP for each individual. A high *D*_*CA*−*OP*_ score can be attained either by giving high CA or low OP. This new approach (*D*_*CA*−*OP*_) would allow researchers to quantify the balance between CA and OP in parenting style. Furthermore, we also found a positive correlation between rGMV in the R-SMG and offspring's originality/fluency scores. Originality is the ability to produce ideas that differ from those of others. According to a recent paper, “the originality of creativity measured by divergent thinking (CMDT) is a unique variable that is positively correlated with psychometric intelligence and other psychological measures” [19]. Our findings showed consistency with previous studies which demonstrated neural evidence that the IPL, the subregions of which are the SMG and AG, may contribute to the production of original ideas [40, 41]. Moreover, a recent study on 23–59-year-old participants suggests that larger volume of right SMG was associated with higher emotion recognition abilities [44].

According to the above results, we indicated that different parenting styles are suitable for offspring with higher SQ (systemizers) or higher EQ (emphatizers). In terms of improving offspring's creativity or originality in thinking, we found that for systemizers, overprotective parenting may not be suitable. On the contrary, overprotective parenting may be appropriate for emphatizers. Further longitudinal research is needed to clarify the causal relationships involved. At the current time, we can only speculate about explanations for this result. The insensitivity of systemizers to their surroundings may lead them to focus more on their thoughts so that they tend to be able to solve the problems they face alone. Systemizers might not necessarily experience the distressing emotions of others and tend to be neglectful of social information for understanding the causes of human behavior [45]. High systemizers also tend to seek problems about which they can think long and hard but are less inclined to actively use deliberative thinking to understand others' perspectives [46]. Therefore, overprotective parenting or an excessive number of interventions may not be appropriate because it could disrupt the offspring's focus and creativity in solving the problems they are facing. In contrast, empathizers are vulnerable to their surroundings, are often interested in others' emotions, and intuitively understand others' mental states [47]. High empathizers are also more sensitive to others' reactions [48], so they may absorb emotions of other people and unconsciously compare themselves to those around them, leading to a lack of confidence in their abilities. At this time, parental intervention in terms of overprotective parenting may be needed. Parents are expected to motivate and convince their offspring that they have the creativity to solve their own problems.

Our findings about the significant and positive correlation between the volume of R-SMG and offspring's creativity also showed consistency with some previous studies. A previous fMRI study demonstrated that angular and SMG are associated with creative thinking [49]. Additionally, a previous positron emission tomography (PET) study also pointed out the relationship between SMG and creative tasks especially in terms of imagination process and flexibility in thinking [50]. The relationship between the SMG and creativity has also been described specifically in previous studies, such as its role in decision-making with low error rates [51], in imagination [52], in switching tasks [50, 53, 54], and in planning for task solving [55]. These previous studies support our finding that the observed greater SMG volumes may account for greater creativity.

In this study, we evaluated offspring's creativity using DT tests (named CMDT). DT has been reported to be associated with creativity [4]. Moreover, intelligence test scores showed a weaker relationship with CMDT than creativity [56]. DT tests consist of four subscales: fluency, flexibility, originality, and elaboration. Our findings demonstrated a specific positive correlation between originality and rGMV in the R-SMG. R-SMG is part of the posterior parietal cortex (PPC) [57]. Many studies have assessed the PPC's contribution to DT. Several previous studies describe a stronger relationship between PPC and originality as dimension of DT rather than fluency [58–61]. On the other hand, other publications showed a significant relationship between prefrontal area and fluency as dimension of DT [62–65]. On the basis of these previous studies, it is possible that the observed greater R-SMG volumes may account for the greater creativity, particularly in the dimension of originality.

To sum up, using whole-brain VBM with a large number of participants, we found significant interactive effects between parenting style and offspring's E-S cognitive style on rGMV in the right SMG. Consistent with the previous studies, we also found that the rGMV in the right SMG was significantly and positively correlated with the offspring's originality/fluency scores, a dimension of creativity. In terms of appropriate parenting style, high care but not an overprotective parenting style may be suitable for offspring with higher SQ. Overprotective parenting or excessive intervention may not be suitable because it may interfere with the offspring's focus and creativity in solving the problems he is facing. In contrast, the overprotective parenting style may be appropriate for offspring with higher EQ. Due to their sensitiveness to others' emotions, parental intervention in terms of overprotective parenting may be needed. Parents are expected to motivate and convince their offspring that they have the creativity to solve their own problems.

## 5. Limitation

Our study has several limitations. First, our findings are based on a cross-sectional design, meaning that although we demonstrated the interactive effect between parenting style and the offspring's E-S cognitive style, we could not confirm the causal relationship. Second, we used retrospective self-reported measurements of parenting style, which are prone to memory and self-preference biases. A more objective record of parental behaviors will help reduce the influence of potential confounding factors.

## 6. Conclusion

In this study, we found significant interactive effects between parenting style and offspring's E-S cognitive style on rGMV in the right SMG, which significantly and positively correlated with the offspring's originality/fluency scores.

## 7. Future Work

Longitudinal studies will be necessary to extend our cross-sectional findings and should be undertaken in future studies. Future studies should also investigate the biological mechanisms underlying the interaction between parenting style and offspring's E-S cognitive style on rGMV in the right SMG. Elucidating this issue may be helpful for understanding the mechanisms underlying the current results. We hope this research can provide critical information for parents regarding the parenting style that suits their offspring's E-S cognitive styles to improve the offspring's creativity and quality of life.

## Data Availability

The datasets analyzed during the current study can be provided to third parties by contacting the corresponding author. Any request must be approved by the Ethics Committee of Tohoku University, School of Medicine.
